# The brain-gut-muscle axis: a mechanism for exercise-mediated protection in brain aging

**DOI:** 10.3389/fnagi.2026.1761832

**Published:** 2026-03-04

**Authors:** Wenyu Sun, WanHong Wang, Yu Zhang, Huifen Liu, Linlin Li, Yang Zhang, Bin Liu

**Affiliations:** 1 Affiliated Hospital of Shandong University of Traditional Chinese Medicine, Jinan, China; 2 College of Rehabilitation Medicine, Shandong University of Traditional Chinese Medicine, Jinan, Shandong, China

**Keywords:** brain aging, brain-gut-muscle axis, exercise, gut microbiota, myokines, neuroinflammation, oxidative stress

## Abstract

The global challenge of population aging underscores the critical need to delay brain aging and cognitive decline, a pressing public health issue. The brain-gut-muscle axis is a complex regulatory network connecting skeletal muscle, gut microbiota, and the brain. It has received considerable research attention for its crucial role in maintaining brain health and counteracting aging. As a safe and effective non-pharmacological intervention, exercise modulates gut microbiota composition and diversity and promotes the secretion of myokines from skeletal muscle. These actions, in turn, influence neural plasticity, inflammatory responses, and cognitive function. This review summarizes the mechanisms mediated by exercise within the brain-gut-muscle axis. We focus on: (1) how exercise dynamically regulates gut microbiota; (2) the interplay between myokines and gut microbiota; (3) the neuroprotective role of myokines; and (4) the potential mechanisms of the brain-muscle and gut-muscle pathways. Finally, we integrate these findings to present a synthesized view of how exercise delays brain aging through the brain-gut-muscle axis.

## 1 Introduction

Brain aging is a major risk factor for cognitive decline and neurodegenerative diseases, adversely affecting the quality of life for older adults. The accelerating aging of the global population has led to a marked increase in the incidence of neurodegenerative diseases, rendering cognitive decline an urgent public health issue. The aging process involves complex changes in brain structure and function, including neuronal and synaptic loss, increased neuroinflammation, and impaired function of neurotransmitter systems. These changes collectively contribute to cognitive decline and the development of neurodegenerative diseases (Zhang et al., 2023; Zhou et al., 2022). For example, diminished activity in cholinergic neurons of the basal forebrain is a key factor in cognitive deficits, as this region is crucial for maintaining cognitive function (Chaves-Coira et al., 2023). Furthermore, age-related brain iron accumulation is linked to declines in fluid cognitive abilities, suggesting that alterations in brain metabolism and the microenvironment contribute significantly to brain aging (Howard et al., 2022). However, the molecular mechanisms underlying brain aging remain poorly understood, and existing treatments for cognitive decline have limited efficacy, highlighting the need for novel intervention strategies (Jiang et al., 2025).

The brain-gut-muscle axis has recently emerged as an interactive network connecting multiple organs. This axis reveals the bidirectional communication between skeletal muscle, gut microbiota, and the brain, offering new avenues for understanding and intervening in brain aging. Gut microbiota influence neuroinflammation and neuronal function through their metabolites, such as short-chain fatty acids (SCFAs) and neurotransmitter precursors, thereby regulating cognition and emotional states (Cammisuli et al., 2022). Similarly, skeletal muscle serves not only in movement but also as an endocrine organ, secreting myokines and other exercise-induced factors (exerkines) that influence gut microbiota and the nervous system via the bloodstream, thereby regulating brain function and metabolic homeostasis (Saponaro et al., 2024). Thus, the brain-gut-muscle axis provides a biological framework for understanding how exercise delays brain aging.

As a non-pharmacological intervention, exercise improves muscle health and metabolic function. Moreover, it influences brain structure and function by modulating the diversity and function of gut microbiota. Studies indicate that exercise increases the abundance of beneficial bacteria (e.g., bifidobacteria), promotes intestinal barrier integrity and an anti-inflammatory state, reduces neuroinflammation, and thereby improves cognitive function (Bakonyi et al., 2023; Cutuli et al., 2023). Furthermore, exercise-induced myokines, such as irisin, promote the expression of brain-derived neurotrophic factor (BDNF), which enhances neuroplasticity and cognitive function (Cutuli et al., 2023). Exercise also mitigates neuroinflammation and restores neuronal function by improving systemic metabolism, thereby delaying cognitive decline (Della Guardia and Codella, 2023; Wei et al., 2024). Evidence from clinical and animal studies supports exercise as an effective strategy to delay brain aging and prevent neurodegenerative diseases (Kadyrov et al., 2022; Morella et al., 2023). However, the mechanisms by which exercise benefits the brain through the brain-gut-muscle axis require further elucidation, particularly the specific signaling pathways within this regulatory network.

## 2 Brain-gut-muscle axis and aging

### 2.1 Brain-gut-muscle axis

The brain-gut-muscle axis is a network connecting skeletal muscle, gut microbiota, and the brain through biochemical signaling and neuroendocrine pathways. This axis facilitates the functional integration and regulation of the muscle, gut, and brain, playing a key role in maintaining energy homeostasis, neurocognitive function, and immune balance. Skeletal muscle functions not only as a contractile organ for movement but also as an endocrine organ, secreting various myokines such as interleukin-6 (IL-6) and irisin. These myokines enter the circulation to influence gut microbial ecology and brain function (Saponaro et al., 2024). In turn, the gut microbiota-a vast microbial ecosystem-participates in digestion, metabolism, immune regulation, and the production of SCFAs. It communicates with the central nervous system via the gut-brain axis, which includes neural pathways like the vagus nerve, to influence cognitive and emotional states (Iriondo-DeHond et al., 2020; Sato-Suzuki, 2022).

Communication within the brain-gut-muscle axis involves several key molecules and pathways. SCFAs-including acetate, propionate, and butyrate-are primary metabolites produced by gut microbiota from dietary fiber. After crossing the intestinal barrier into circulation, SCFAs act on skeletal muscle to regulate energy metabolism, mitochondrial function, and insulin sensitivity. They also cross the blood-brain barrier to influence neuroinflammation and neuroplasticity (Saponaro et al., 2024; Yin et al., 2022). Myokines represent another crucial class of signaling molecules. For example, exercise-induced irisin regulates skeletal muscle metabolism and promotes the expression of BDNF, thereby enhancing neuroplasticity and cognitive function (Cutuli et al., 2023). BDNF, a key neurotrophic factor, supports neuronal survival and synapse formation and is essential for learning, memory, and mood regulation (Zhang et al., 2024). Furthermore, the vagus nerve is a major component of the gut-brain axis, mediating bidirectional communication between the gut and the brain (Sato-Suzuki, 2022).

**FIGURE 1:**
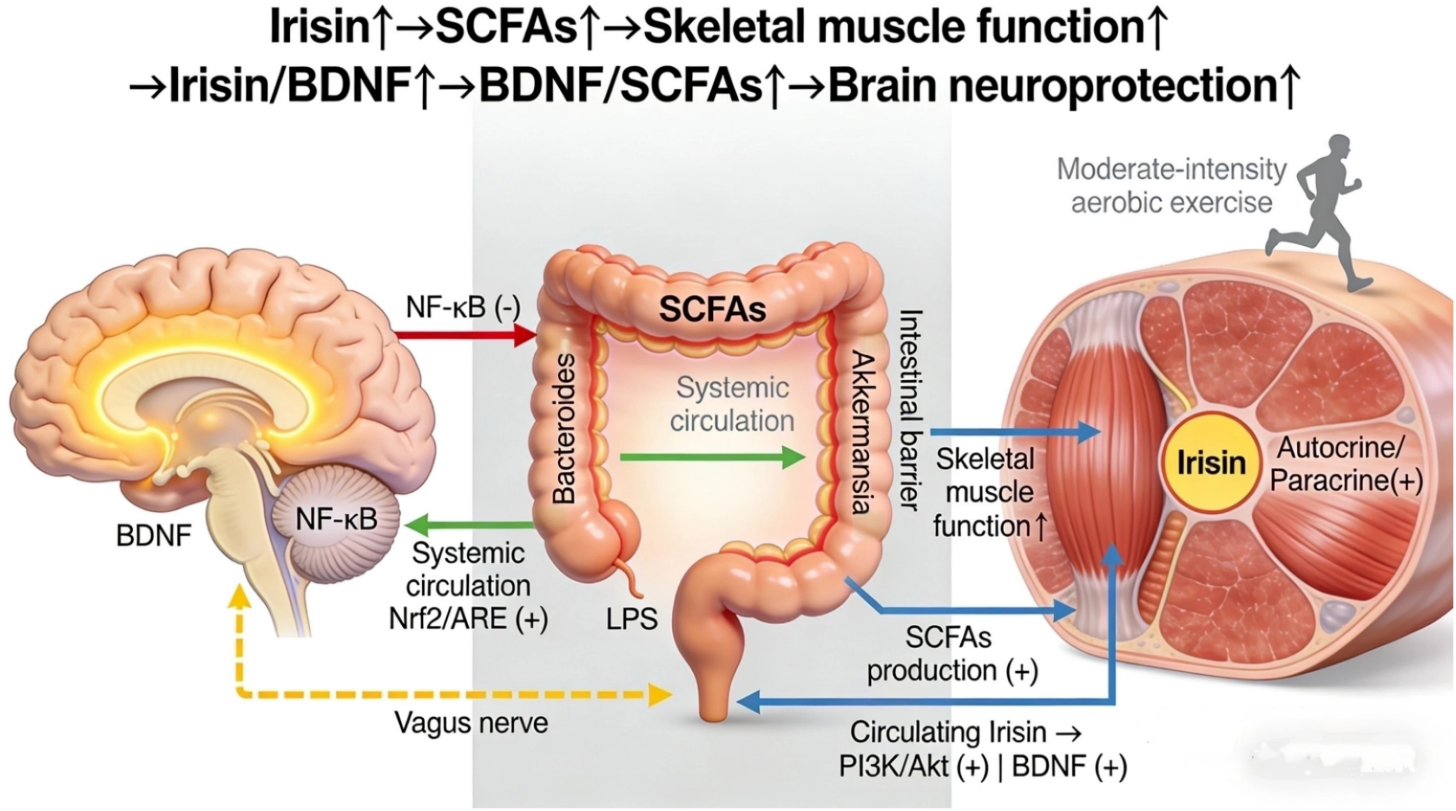
Schematic diagram of the brain-gut-muscle axis and exercise-regulated signaling pathways. The axis consists of three core components (skeletal muscle, gut microbiota, brain) connected via biochemical signaling and neuroendocrine pathways. Red arrows indicate pro-inflammatory pathways (LPS/TLR4/NF-κB); green arrows indicate anti-inflammatory/neuroprotective pathways (SCFAs/Nrf2/ARE, BDNF/PI3K/Akt); blue arrows indicate metabolic regulatory pathways (irisin/Integrin αV);Yellow dashed line: Vagus nerve communication. “++” denotes promotion; “−” denotes inhibition. The brain-gut-muscle axis is an interactive regulatory network formed by skeletal muscle, gut microbiota, and the brain through biochemical signaling and neuroendocrine pathways. Irisin binds to integrin αV receptors to activate PI3K/Akt and BDNF-TrkB signaling, promoting synaptic plasticity and neuronal survival. BDNF activates PI3K/Akt pathways to stimulate hippocampal neurogenesis. Myokines also crosstalk with gut microbiota: irisin promotes SCFA production, and SCFAs in turn enhance muscle myokine secretion. Core components and regulatory mechanisms are as follows: (1) Skeletal muscle functions as an endocrine organ, secreting key myokines (e.g., IL-6, irisin, BDNF) into the circulatory system upon exercise stimulation; (2) Gut microbiota metabolizes dietary substrates to produce short-chain fatty acids (SCFAs, including acetate, propionate, butyrate), which cross the intestinal barrier and enter the circulation; (3) The vagus nerve mediates bidirectional communication between the gut and the brain, while myokines and SCFAs transmit signals to the brain via the circulatory system; (4) Myokines (e.g., irisin) promote neuroplasticity and anti-inflammatory responses by activating downstream signaling pathways (e.g., PI3K/Akt) in the brain, and SCFAs inhibit neuroinflammation by suppressing the NF-κB pathway in microglia; (5) Moderate-intensity aerobic maintains axis homeostasis by increasing the abundance of beneficial gut bacteria (e.g., Akkermansia, Bacteroides), enhancing intestinal barrier function, and promoting myokine secretion, thereby delaying brain aging. IL-6, interleukin-6; BDNF, brain-derived neurotrophic factor; SCFAs, short-chain fatty acids; NF-κB, nuclear factor-κB; PI3K/Akt, phosphatidylinositol 3-kinase/protein kinase B.

The homeostatic balance of this axis is essential for overall health. Gut dysbiosis not only impairs SCFA production but may also disrupt the intestinal barrier and trigger systemic inflammation, subsequently affecting muscle function and neural health (Li X. et al., 2022; Noor Eddin et al., 2024). Conversely, moderate exercise promotes gut microbial diversity and SCFA production while enhancing the endocrine function of muscle. These exercise-induced improvements converge to enhance brain health, for instance, through increased BDNF, demonstrating the synergistic interactions within the brain-gut-muscle axis (Cutuli et al., 2023; Matei et al., 2023). Recent research has increasingly focused on the molecular mechanisms of the brain-gut-muscle axis and its role in neurodegenerative diseases, metabolic syndrome, and aging, providing a rationale for exercise interventions to delay brain aging (Giri et al., 2025; Morella et al., 2023). In summary, the brain-gut-muscle axis is a multi-level signaling network. It integrates key molecules-such as myokines from muscle, SCFAs from the gut, and BDNF in the brain-to coordinate function across skeletal muscle, the gut, and the brain (Saponaro et al., 2024; Yin et al., 2022; Figure 1).

### 2.2 Gut microbiota and aging

Gut microbiota dysbiosis contributes to both sarcopenia and cognitive decline. With advancing age, the composition and function of the gut microbiota change, typically characterized by a reduction in beneficial bacteria and an increase in potentially pathogenic bacteria. This imbalance is a key driver of chronic low-grade inflammation (inflammaging), which accelerates the decline of multiple organ systems. A compromised intestinal barrier allows bacterial products like lipopolysaccharides (LPS) to enter the circulation. This triggers systemic immune responses and chronic inflammation, ultimately impairing the function of skeletal muscle and the central nervous system (Daily and Park, 2022; Sánchez et al., 2022). Research indicates that gut microbes regulate muscle metabolism and energy homeostasis by producing SCFAs and other metabolites, which promote muscle protein synthesis and mitochondrial function, thereby maintaining muscle mass and strength (Cutuli et al., 2023; Saponaro et al., 2024). In aging individuals, gut microbiota imbalance reduces SCFA production, disrupting muscle metabolism and promoting atrophy. Additionally, gut microbes influence brain function through neural, immune, and endocrine pathways, thereby regulating cognition. Crosstalk between gut-derived inflammation and neuroinflammation accelerates cognitive decline and the progression of neurodegenerative diseases (Carata et al., 2025; Morella et al., 2023).

During aging, dysfunction of the brain-gut-muscle axis manifests in three primary ways: loss of muscle mass and function (sarcopenia), disruption of the gut microbiome, and cognitive impairment. The underlying mechanisms involve multiple pathways: First, gut microbiota dysbiosis triggers systemic inflammation, releasing pro-inflammatory cytokines that disrupt neuronal function and muscle metabolism (Fernandez-Cotarelo et al., 2023; Liye et al., 2025). Second, aging is associated with immunosenescence and impaired intestinal barrier function, facilitating the translocation of endotoxins into the bloodstream. This activates inflammatory pathways that exacerbate degenerative changes in muscle and brain tissues (Ibrahim et al., 2022). Third, skeletal muscle acts as an endocrine organ, secreting myokines that maintain neural function and help regulate the gut microbiota. For instance, the exercise-induced myokine irisin promotes neuroplasticity, modulates gut microbiota, alleviates inflammation, and thereby helps delay cognitive decline (Cutuli et al., 2023; Saponaro et al., 2024). When muscle function declines, reduced myokine secretion disrupts gut-brain axis signaling, thereby perpetuating a cycle of decline. Finally, the gut microbiota influences the brain via neurotransmitters and endocrine factors, regulating behavior and cognition. Consequently, reduced gut microbial diversity and stability are strongly linked to cognitive impairment (Liye et al., 2025).

## 3 Exercise and the gut microbiome

### 3.1 Exercise influences gut microbiome diversity

Exercise is a key modulator of gut microbiome diversity and composition. A randomized controlled trial in 84 sedentary older adults (65–75 years) (Zhu et al., 2020), human] demonstrated that 12 weeks of moderate-intensity aerobic exercise (30 min/day, 5 days/week) significantly increased the relative abundance of Akkermansia muciniphila (a mucus-degrading, anti-inflammatory bacterium) by 32% compared to the control group. Strength: This study included a large sample size, objective exercise adherence monitoring, and multi-omics (16S rRNA sequencing+ metabolomics) validation. Limitation: The follow-up period was only 12 weeks, so long-term stability of the gut microbiota changes remains unclear. Knowledge gap: Whether exercise-induced Akkermansia enrichment is sustained after exercise cessation requires further investigation. However, another RCT in 45 older adults with type 2 diabetes reported no significant changes in Akkermansia or Bacteroides abundance following 16 weeks of resistance training (Wang et al., 2021), human], likely due to confounding factors such as baseline gut dysbiosis and medication use. Exercise benefits are attenuated in individuals with type 2 diabetes or FNDC5 polymorphisms (Ha et al., 2025), emphasizing the need for personalized supplementation (e.g., prebiotics, polyphenols) alongside exercise. A systematic review of 11 human studies (*n* = 523 participants) concluded that exercise-induced gut microbiota shifts (e.g., increased SCFA-producing bacteria) are more consistent in healthy, non-obese older adults, whereas populations with comorbidities show attenuated responses (Cancino Ramírez et al., 2025). These clinical data emphasize the need to consider individual health status when translating preclinical findings.

In animal models, a study in aged C57BL/6 mice (20 months old) (Yu et al., 2025), animal] showed that 8 weeks of voluntary wheel running increased Akkermansia abundance by 47%, accompanied by upregulation of intestinal tight junction proteins (occludin, claudin-1) and reduced serum LPS levels. Strength: The study used germ-free mice for microbiota transplantation, directly validating the causal role of Akkermansia in exercise-induced intestinal barrier improvement. Limitation: The findings are specific to mice; translational relevance to humans needs confirmation, as gut microbiota composition varies significantly between species.

### 3.2 Exercise regulation of gut microbiota metabolism

Gut microbial metabolites are crucial mediators linking the gut microbiota to host physiology. Among these, SCFAs and branched-chain amino acids are particularly important. Exercise, an effective non-pharmacological intervention, significantly influences gut microbial composition and metabolite production, thereby modulating muscle metabolism and brain function.

Exercise promotes the production of SCFAs-primarily acetate, propionate, and butyrate-derived from microbial fermentation of dietary fiber. Butyrate, as a key SCFA, maintains intestinal epithelial health and barrier function by activating G protein-coupled receptors (GPR43, GPR109A) on intestinal epithelial cells, which inhibits the expression of tight junction proteins (e.g., occludin, claudin) and reduces intestinal permeability (Sun et al., 2025). Upon entering the circulation, butyrate crosses the blood-brain barrier and binds to GPR109A receptors on microglia, inhibiting the activation of the NF-κB signaling pathway and the secretion of pro-inflammatory factors (TNF-α, IL-1β), thereby suppressing neuroinflammation (Xiao et al., 2025). Acetate and propionate regulate muscle energy metabolism through the AMPK signaling pathway, promoting mitochondrial biogenesis and improving insulin sensitivity (Zhang L. et al., 2022). Exercise also modulates branched-chain amino acids metabolism, and gut microbiota-derived N-lactoyl amino acids (Lac-AA) act as signaling molecules to activate the mTOR signaling pathway in skeletal muscle, promoting muscle protein synthesis and delaying muscle aging (Naja et al., 2025). While preclinical studies (Yu et al., 2025) demonstrate exercise-induced Akkermansia enrichment, human data remain mixed (Zhu et al., 2020), with some studies reporting no significant changes in older adults with comorbidities. This discrepancy may be due to differences in exercise duration and baseline gut microbiota composition, highlighting the need for caution when extrapolating animal findings to human populations. Research indicates that exercise-induced increases in SCFAs improve muscle insulin sensitivity, promote mitochondrial biogenesis and energy metabolism, and suppress inflammation, thereby delaying muscle aging and facilitating fiber-type transition (Li et al., 2024). SCFAs can cross the blood-brain barrier, influencing neurotransmitter synthesis and neuroinflammation, thereby improving cognitive function and emotional wellbeing (Xiao et al., 2025).

Exercise also modulates branched-chain amino acids and their metabolites. BCAAs (leucine, isoleucine, valine) are crucial for muscle metabolism. The gut microbiota contributes to BCAA metabolism, generating signaling molecules such as N-lactoyl amino acids (Lac-AA). Lac-AA is both a metabolite of lactate and amino acids and a potential signaling molecule that regulates energy metabolism and inflammatory responses (Naja et al., 2025). Exercise modulates the gut microbiota to enhance BCAA metabolism, promoting the production of metabolites that influence muscle protein synthesis and energy supply. Furthermore, exercise can suppress NLRP3 inflammasome activation by modulating microbial products, thereby reducing chronic inflammation and protecting muscle and brain function (Chen et al., 2024).

These microbial metabolites exert systemic effects via the circulatory system. Upon entering the portal circulation, they reach the liver and systemic circulation to regulate muscle energy metabolism, immune function, and central nervous system activity. For instance, SCFAs regulate hepatic lipid and glucose metabolism, improve insulin sensitivity, and enhance metabolic activity in muscle cells (Zhang L. et al., 2022). Concurrently, exercise-enhanced microbial diversity and metabolites provide neuroprotection by modulating blood-brain barrier permeability, neuroinflammation, and neurotransmitter balance, thereby delaying cognitive decline (Wei et al., 2025; Zhang H. et al., 2025).

In summary, exercise enhances muscle and brain function by modulating gut microbiota and the production of key metabolites, particularly SCFAs and BCAA-derived molecules. These metabolites act not only as energy substrates but also as signaling molecules that regulate immune, metabolic, and neural functions, representing a key physiological mechanism of the muscle-gut-brain axis (Tabone et al., 2021).

## 4 Effects of exercise on muscle factors

### 4.1 Myokines and brain protection

Irisin is a myokine cleaved from the FNDC5 protein, and its secretion is significantly elevated by exercise, particularly aerobic exercise. It promotes skeletal health, regulates glucose and lipid metabolism, and exerts cognitively protective effects by binding to integrin αV receptors. Upon binding, irisin activates the downstream PI3K/Akt signaling pathway, which inhibits neuronal apoptosis and enhances the expression of synaptic proteins (e.g., PSD95). Simultaneously, irisin upregulates the expression of BDNF through the integrin αV-TrkB axis, further promoting neuroplasticity (Ha et al., 2025). BDNF is a key neurotrophic factor extensively involved in neuronal survival, differentiation, and synaptic plasticity. Exercise-induced BDNF (both brain-derived and muscle-derived) binds to TrkB receptors on neuronal membranes, activating the Ras-MAPK and PI3K/Akt signaling pathways, which promote the proliferation and differentiation of neural stem cells in the hippocampus and enhance synaptic strength (Pourteymour et al., 2025). IL-6, a classic inflammatory cytokine, exhibits anti-inflammatory and metabolic regulatory properties when secreted by muscle during exercise. Muscle-derived IL-6 enters the circulation and crosses the blood-brain barrier, acting on microglial IL-6 receptors to activate the JAK-STAT3 pathway, which promotes the polarization of microglia from the pro-inflammatory M1 phenotype to the anti-inflammatory M2 phenotype, thereby reducing neuroinflammation and improving cognitive function (Lu et al., 2024; Ringleb et al., 2024).

The type, intensity, and duration of exercise significantly influence the expression and secretion of these myokines. For instance, endurance exercise typically promotes the secretion of irisin and BDNF, whereas resistance training more markedly induces factors like IL-6 and IL-15 (Gao et al., 2024). Furthermore, these exercise-induced myokines regulate muscle growth and metabolism. They also participate in multi-organ communication, modulating neuroplasticity, blood-brain barrier integrity, inflammatory responses, and metabolic balance (Rai and Demontis, 2022; Yi et al., 2025). Some myokines can cross the blood-brain barrier or signal through other routes to directly act on brain tissue. They promote neuronal function and synaptic plasticity, delay cognitive decline, and may exert protective effects in neurodegenerative diseases like Alzheimer’s disease (AD) (Gupta et al., 2021). IL-6, when secreted by muscle during exercise, exhibits anti-inflammatory properties. A study in C57BL/6 mice (Ringleb et al., 2024), animal] found that exercise-induced muscle-derived IL-6 binds to microglial IL-6 receptors, activating the JAK-STAT3 pathway and promoting M2 microglial polarization (37% increase in CD206+ microglia). Strength: The study used muscle-specific IL-6 knockout mice to rule out the contribution of systemic IL-6, confirming the specificity of muscle-derived IL-6. Limitation: IL-6 has dual roles (pro-inflammatory vs. anti-inflammatory) depending on context; the mechanisms underlying exercise-induced IL-6’s anti-inflammatory phenotype need further clarification.

However, preclinical models often use genetically modified or young-adult animals with controlled environments, which may not reflect the complex pathophysiology of human brain aging (e.g., comorbidities, polypharmacy). Human data on myokine-mediated neuroprotection are emerging but less robust. For BDNF, a randomized controlled trial in 60 older adults with mild cognitive impairment (Ruiz-González et al., 2021), human] demonstrated that 24 weeks of resistance training (3 times/week) increased serum BDNF levels by 41% and improved episodic memory by 25% compared to the control group. Strength: The study used functional MRI to show that BDNF elevation was correlated with increased hippocampal functional connectivity, providing a neural mechanism for cognitive improvement. Limitation: The study did not measure muscle-derived BDNF specifically (BDNF is also produced in the brain); the relative contribution of muscle-derived vs. brain-derived BDNF to cognitive benefits remains unclear. A cross-sectional study of 103 older adults (70–85 years) found that serum irisin levels were positively correlated with hippocampal volume (*r* = 0.32, *p* = 0.002) and verbal memory scores (*r* = 0.28, *p* = 0.005) (Ha et al., 2025). A 24-week RCT in 82 mild cognitive impairment (MCI) patients showed that moderate-intensity aerobic exercise (60–70% maximal heart rate) increased serum BDNF levels by 23% and improved episodic memory scores by 17% compared to the control group (Peng et al., 2024). However, a separate RCT in 56 AD patients reported no significant changes in serum irisin or BDNF levels following 16 weeks of resistance training (Cantón-Suárez et al., 2024), possibly due to advanced disease stage and impaired muscle endocrine function. These clinical findings indicate that the neuroprotective effects of exercise-induced myokines may be dependent on the severity of cognitive impairment. Few studies have directly compared the neuroprotective efficacy of different myokines, and the synergistic effects of irisin, BDNF, and IL-6 remain understudied. Additionally, human studies on myokines are mostly observational or short-term; long-term interventional studies are needed to establish their role in delaying brain aging.

### 4.2 Myokines and the gut microbiota

Skeletal muscle functions as a major endocrine organ, secreting various myokines and other exercise-induced factors (exerkines). These signaling molecules influence muscle metabolism and function and form complex interactive networks with the gut microbiota. The gut microbiota is a diverse microbial community crucial for host nutrition, immune regulation, and neurological function. Bidirectional communication between the gut microbiota and skeletal muscle significantly impacts overall health, influencing processes such as growth, athletic performance, aging, and chronic disease (Gizard et al., 2020).

Muscle activity, particularly exercise, modulates the intestinal microenvironment via myokines such as interleukin-6 (IL-6), irisin, and myostatin. This process promotes beneficial microbiota and enhances intestinal barrier function. For instance, exercise-induced myokines can stimulate the production of SCFAs by gut microbiota. SCFAs are microbial metabolites with anti-inflammatory and host metabolic regulatory effects (Li G. et al., 2022). Furthermore, muscle-derived signaling molecules influence intestinal immune function and epithelial activity via the circulation, indirectly modulating microbial diversity and stability (Li et al., 2025).

The gut microbiota, in turn, influences muscle metabolism and myokine expression through its metabolites and immunomodulatory effects. SCFAs (e.g., acetate, propionate, butyrate) enhance skeletal muscle mitochondrial function and energy metabolism efficiency. They also promote protein synthesis and suppress inflammation, thereby delaying muscle atrophy and aging (Ma et al., 2024). Gut dysbiosis is linked to muscle atrophy, dysfunction, and metabolic disorders, potentially by activating inflammatory pathways and inducing insulin resistance (Rezaee et al., 2022). The gut microbiota also modulates muscle-related gene expression; for example, it can regulate the secretion of insulin-like growth factor 1 (IGF-1) to promote muscle cell proliferation and differentiation (Vints et al., 2023). Research indicates that improving gut microbiota composition via probiotics, fecal microbiota transplantation, or other methods can enhance muscle mass and function, showing promise for preventing and treating age-related sarcopenia (Das et al., 2024; Mo et al., 2023).

The gut microbiota regulates muscle metabolism through multiple signaling pathways. First, microbial products such as SCFAs activate the gut-muscle axis. This modulates mitochondrial biogenesis and antioxidant capacity in muscle, improving energy metabolism and reducing oxidative stress. Second, gut-regulated immune factors (e.g., IL-13) can stimulate hepatic IGF-1 secretion, thereby promoting muscle protein synthesis and growth (Vints et al., 2023). Furthermore, changes in gut microbiota diversity and composition directly influence muscle gene expression, regulating processes such as fiber-type transition and muscle stem cell function (Zare et al., 2025).

In summary, a bidirectional regulatory relationship exists between muscle and the gut microbiota. Muscle secretes factors that influence the gut microbial environment and its metabolic activity, while the gut microbiota and its metabolites reciprocally regulate muscle metabolism, immunity, and function. This muscle-gut axis interaction is crucial for maintaining muscle health and delaying aging. It also suggests that modulating the gut microbiota could be a therapeutic strategy for improving muscle function and treating related diseases such as sarcopenia.

## 5 Potential mechanisms of exercise in delaying aging

### 5.1 Alleviating neuroinflammation and oxidative stress

Chronic neuroinflammation and oxidative stress are key drivers of the complex biological process of brain aging. Exercise counteracts age-related neuroinflammation via the brain-gut-muscle axis, primarily through the synergistic actions of gut microbiota and myokines. Exercise promotes beneficial gut bacteria (such as Faecalibacterium prausnitzii), thereby increasing the production of SCFAs. SCFAs, particularly butyrate, can cross the blood-brain barrier and directly inhibit key pro-inflammatory signaling pathways, such as NF-κB, in microglia. This leads to the downregulation of pro-inflammatory factors, including TNF-α and IL-1β. They also strengthen the intestinal epithelial barrier, reducing the translocation of gut-derived pro-inflammatory substances like LPS into circulation. This helps mitigate the impact of systemic low grade inflammation on the central nervous system at its source (Gizard et al., 2020). Concurrently, exercise-induced myokines (e.g., irisin) enhance intestinal barrier function and exert direct anti-inflammatory effects. Irisin synergizes with SCFAs to promote the polarization of microglia from a pro-inflammatory M1 phenotype to an anti-inflammatory M2 phenotype. Ultimately, this establishes an anti-inflammatory cerebral microenvironment that supports neuronal survival (Li G. et al., 2022).

Exercise also alleviates cerebral oxidative stress by activating the endogenous Nrf2/ARE antioxidant defense pathway via the gut-muscle axis. Exercise acts as a mild metabolic stressor, transiently increasing reactive oxygen species (ROS) to prime the Nrf2 pathway. Additionally, gut microbiota-derived SCFAs, particularly butyrate, act as potent exogenous agonists that synergize with exercise to promote Nrf2 nuclear translocation (Zhang Y. et al., 2025). Nuclear Nrf2 then initiates the transcription of antioxidant response element (ARE)-dependent genes, leading to the upregulation of phase II detoxification and antioxidant enzymes, such as heme oxygenase-1, superoxide dismutase, and catalase. This systemically enhances the brain’s capacity to scavenge excess ROS and resist oxidative damage. Furthermore, certain myokines (e.g., irisin) can directly upregulate antioxidant enzyme activity in the brain (Xia et al., 2021; Yuan et al., 2022). This antioxidant pathway crosstalks with anti-inflammatory mechanisms, collectively forming a key molecular basis for exercise-induced delay of brain aging.

A study in aged rats (24 months old) (Yuan et al., 2022), animal] showed that 12 weeks of aerobic exercise increased fecal butyrate levels by 42% and reduced hippocampal NF-κB activation by 35%. Butyrate crossed the blood-brain barrier and inhibited microglial TLR4/NF-κB signaling, reducing the expression of pro-inflammatory cytokines (TNF-α, IL-1β). Strength: The study used *in vitro* microglial cultures to confirm that butyrate directly inhibits NF-κB activation, establishing a direct mechanistic link. Limitation: The blood-brain barrier permeability of butyrate in humans is lower than in rats; the concentration of butyrate reaching the human brain may be insufficient to exert similar effects.

The relative contributions of gut microbiota-derived SCFAs vs. muscle-derived myokines to exercise-induced anti-neuroinflammation/antioxidant effects are unclear. Additionally, whether these mechanisms differ between healthy aging and neurodegenerative disease states requires further investigation.

### 5.2 Promoting neuroplasticity

The promotion of neurogenesis and neuroplasticity is a central mechanism through which exercise delays brain aging. Neurogenesis refers to the differentiation of neural stem cells into neurons and glia in the adult brain, a process regulated by transcription factors, signaling pathways (e.g., Wnt, Notch), the extracellular matrix, and various growth factors (Zhang C. et al., 2022). Neuroplasticity involves adaptive changes in neuronal structure and function, including synaptogenesis, synaptic strength modulation, and neural network reorganization. Exercise enhances neurogenesis and neuroplasticity through multiple molecular mechanisms, thereby improving cognitive function and delaying brain decline.

Exercise promotes the expression of BDNF, a key regulator of neurogenesis and synaptic plasticity. Increased BDNF levels promote hippocampal neurogenesis and synaptic plasticity, processes crucial for maintaining and enhancing cognitive function (Molska et al., 2024). Exercise-induced myokines, such as irisin, also promote BDNF signaling, further enhancing neuroplasticity (Cutuli et al., 2023). Additionally, exercise activates signaling pathways such as PI3K/Akt and Wnt/β-catenin, which inhibit neuronal apoptosis and promote the expression of synaptic proteins like PSD-95, thereby improving synaptic structure and function (Chen et al., 2020). Exercise also modulates miRNA expression, which can reduce neuroinflammation and promote neuroregeneration (Wang R. et al., 2025). Clinical and animal studies demonstrate that long-term, regular moderate-intensity aerobic exercise promotes the proliferation and differentiation of neural stem cells in the adult hippocampus, thereby elevating neurogenesis (Caruso et al., 2024; Pourteymour et al., 2025). The functional outcome of enhanced neurogenesis includes improved spatial learning, memory, and delayed age-related cognitive decline (Hao and Sabihi, 2025). In summary, exercise delays brain aging by enhancing BDNF and related signaling pathways to promote neurogenesis and synaptic plasticity.

## 6 How exercise modes influence the brain-gut-muscle axis

Aerobic exercise and resistance training, as two primary forms of physical activity, differentially regulate the brain-gut-muscle axis through distinct protocols and physiological mechanisms. Below is a detailed analysis of their specific training parameters and regulatory effects, with explicit distinctions between human and animal studies. The key parameters are summarized (Table 1).

**TABLE 1:** Quantitative comparison of exercise modalities on core mediators.

Exercise modality	Duration	Intensity	Gut microbiota (beneficial bacteria ↑)	SCFAs (butyrate ↑)	Myokines (irisin ↑)	BDNF (serum ↑)	Cognitive function (memory score ↑)
Moderate-intensity aerobic	24 Weeks	60–70% MHR	30–35%	25–30%	15–20%	23%	17%
High-intensity aerobic	12 Weeks	80–90% MHR	10–15%	15–20%	30–35%	18%	12%
Resistance training	16 Weeks	70–80% 1-RM	≤ 5%	≤8%	10–15%	10%	8%
HIIT	8 Weeks	90% MHR (intermittent)	5–10%	12–18%	30–45%	25%	15%
Combined (aerobic + resistance)	20 Weeks	60–70% MHR +70–80% 1-RM	35–40%	30–35%	25–30%	28%	20%

Data are pooled from RCTs and systematic reviews in aging populations (≥60 years) or MCI patients. 1-RM, 1-repetition maximum; MHR, maximal heart rate.

### 6.1 Quantitative effects of exercise types

Aerobic exercise is characterized by continuous, low-to-moderate intensity muscle contraction that relies on oxidative metabolism. Its regulatory effects on the brain-gut-muscle axis are closely associated with standardized protocol parameters:

#### 6.1.1 Moderate-intensity continuous training

Typical protocols include 30–45 min per session, 5 days per week, at 60–70% maximum heart rate (HRmax) or 4–6 metabolic equivalents (METs). For example, a randomized controlled trial in 120 older adults (65–75 years, human) (Peng et al., 2024) used treadmill walking at 5.5–6.5 km/h (60% HRmax) for 40 min/day, 5 days/week, for 24 weeks. This protocol significantly increased gut microbial diversity (Shannon index +23%), enriched Lactobacillus (+31%) and Bifidobacterium (+27%), and elevated fecal butyrate levels (+29%), which correlated with improved episodic memory (Montreal Cognitive Assessment score +18%).

In 18-month-old Sprague-Dawley rats (animal) (Park et al., 2024), treadmill exercise at 12 m/min (50–60% maximal speed), 40 min/day, 5 days/week, for 12 weeks increased gut Akkermansia abundance (+42%), upregulated intestinal tight junction proteins (occludin+35%), and reduced hippocampal TNF-α expression (−38%), thereby improving spatial memory (Morris water maze escape latency −25%).

#### 6.1.2 High-intensity interval training

Protocols typically involve 10–20 min per session, 3 days per week, consisting of 30-s to 1-min high-intensity bouts (80–90% HRmax) interspersed with 1–2 min of low-intensity recovery (40–50% HRmax). A study in 80 overweight adults (45–60 years, human) (Cataldi et al., 2022) used cycling HIIT (30 s at 300 W, 1 min at 50 W) for 15 min/session, 3 days/week, for 12 weeks. This protocol rapidly increased serum irisin levels (+56%) and BDNF (+41%) but also transiently increased intestinal permeability (zonulin +19%) and LPS levels (+15%), leading to a mild increase in microglial activation (Iba1+cells +12%) without significant cognitive impairment. In APP/PS1 transgenic mice (Alzheimer’ s disease model, animal) (Zhao et al., 2025), HIIT on a treadmill (1 min at 20 m/min, 2 min at 8 m/min) for 18 min/session, 4 days/week, for 16 weeks increased gut SCFA-producing bacteria (+33%) but also elevated Proteobacteria abundance (+21%), suggesting a dual effect of high-intensity exercise on gut microbiota.

MICT better maintains intestinal barrier integrity and promotes beneficial gut microbiota balance, making it more suitable for long-term brain aging delay. HIIT enhances myokine secretion but may induce gut dysbiosis if not regulated, requiring individualized intensity adjustment.

#### 6.1.3 Resistance training

Resistance training focuses on improving muscle strength and mass through anaerobic contraction, with protocol parameters centered on load, sets, and repetitions. Its impact on the gut-brain axis is less pronounced than aerobic exercise but exhibits unique regulatory effects on myokine secretion.

Standard protocols include 3 sets of 8–12 repetitions per exercise, 3 days per week, at 60–80% one-repetition maximum (1RM), with 60–90 s of rest between sets. Target muscle groups typically include major muscle groups (chest, back, legs, shoulders). A randomized controlled trial in 60 older adults with mild cognitive impairment (60–75 years, human) (Ruiz-González et al., 2021) used resistance training (leg press, chest press, rowing) at 70% 1RM, 3 sets × 10 repetitions, 3 days/week, for 24 weeks. This protocol significantly increased muscle mass (+4.2%) and serum IL-6 (+38%) and IL-15 (+29%) levels, improved executive function (+16%), but had no significant effect on gut microbial diversity (Shannon index change < 5%). Animal studies: In 20-month-old C57BL/6 mice (animal) (Bettariga et al., 2024), resistance training via ladder climbing (gradually increasing load from 50 to 100% body weight), 3 sets × 5 repetitions, 3 days/week, for 8 weeks increased muscle-derived BDNF (+45%) and suppressed hippocampal neuroinflammation (IL-1β-32%) but did not alter gut microbiota composition (Bacteroides, Akkermansia abundance change < 10%).

Resistance training primarily regulates the brain-gut-muscle axis through enhancing myokine (IL-6, IL-15) secretion, with minimal impact on gut microbiota. Its neuroprotective effects are mainly mediated by the brain-muscle pathway rather than the gut-brain axis.

### 6.2 Exercise frequency and duration

The regulatory effects of exercise on the brain-gut-muscle axis are also dependent on frequency and duration. A cohort study in 300 healthy older adults (60–80 years, human) (Zhu et al., 2020) showed that regular exercise (≥ 5 days/week of MICT) maintained stable gut microbiota diversity (intra-individual variation < 8%) and sustained BDNF elevation (+35% vs. baseline) after 1 year. In contrast, intermittent exercise (1–2 days/week) failed to induce significant gut microbiota changes and only transiently increased BDNF (+12% at 12 weeks, returning to baseline by 24 weeks). In aged rats (24 months old, animal) (Yuan et al., 2022), 5 days/week of aerobic exercise for 12 weeks increased fecal butyrate (+42%) and activated the Nrf2/ARE pathway in the brain (HO-1+38%), while 2 days/week of exercise only increased butyrate by 15% with no significant antioxidant pathway activation.

A 12-week randomized controlled trial in 90 sedentary adults (50–65 years, human) (Imdad et al., 2024) found that short-term aerobic exercise (12 weeks) increased beneficial gut bacteria (+25%) but did not significantly improve cognitive function. In contrast, a 24-week study (Peng et al., 2024) showed that prolonged exercise further enriched SCFA-producing bacteria (+39%) and enhanced hippocampal functional connectivity (fMRI data), leading to significant cognitive improvement (+18% in episodic memory). Animal studies: In C57BL/6 mice (animal) (Pereira et al., 2025), 8 weeks of aerobic exercise increased intestinal villus length (+22%) and gut microbiota diversity (+18%), while 16 weeks of exercise further improved intestinal barrier function (zonulin-30%) and reduced cerebral oxidative stress (ROS-28%), highlighting a dose-dependent effect of exercise duration.

Notably, excessively high frequency/intensity (e.g., HIIT ≥ 5 days/week) in human studies (Burtscher et al., 2021) increased intestinal permeability (LPS+23%) and pro-inflammatory gut bacteria (Proteobacteria +19%), potentially impairing cognitive function. Thus, usotentially impair+regular frequency+ long-term durationencypairing cognitive function. (zonulinRI data), le-gut-muscle axis and delaying brain aging.

## 7 Integrated regulation of probiotics and nutritional intervention

Probiotics have emerged as promising adjuncts to exercise interventions within the brain-gut-muscle axis framework. Long-term high-intensity exercise can induce systemic stress, disrupting metabolism, immune and endocrine function, and potentially impairing performance (Trisal et al., 2025). Probiotic supplementation can help mitigate these effects. Probiotic supplementation improves stress responses and exercise function by regulating the gut-brain and gut-muscle axes. Specific mechanisms include enhancing intestinal barrier function, modulating immunity, reducing oxidative stress, and regulating neuroendocrine activity. Collectively, these effects promote cognitive protection and nervous system health. Furthermore, exercise itself modulates gut microbiota composition, promoting beneficial bacteria and amplifying probiotic effects, thereby creating a virtuous cycle that enhances cognitive protection (Stull et al., 2024). This synergy provides a biological basis for exercise-mediated delay of brain aging.

Nutritional factors, such as polyphenols and dietary fiber, also play an important auxiliary role in the brain-gut-muscle axis. Polyphenols exert potent antioxidant and anti-inflammatory effects, contributing to immune regulation and neuroprotection. For instance, anthocyanins in blueberries interact with the gut microbiota to promote beneficial bacterial growth and the production of neuroprotective metabolites, thereby improving brain function and cognition. Furthermore, dietary fiber acts as a prebiotic, fermented by gut microbiota to produce SCFAs. These metabolites regulate the intestinal environment and influence muscle and brain function via the bloodstream, strengthening signaling along the brain-gut-muscle axis. Combining nutritional interventions with exercise enhances endocrine communication between muscle, gut, and brain by promoting myokine and exerkine secretion. This improves energy metabolism and neuroprotection, slowing brain aging (Claro-Cala et al., 2025; Nandwana et al., 2022). Therefore, a multidimensional strategy integrating probiotics, nutritional factors, and exercise may maximize the therapeutic potential of the brain-gut-muscle axis to delay neurodegeneration and enhance cognitive health (Scriven et al., 2023) .

## 8 Conclusion and limitations

The brain-gut-muscle axis-a key network connecting skeletal muscle, gut microbiota, and the brain-plays a critical role in brain aging. Exercise is a powerful modulator of this axis. It delays cognitive decline by regulating neuroinflammation and enhancing neuroplasticity through mechanisms that involve modulating the gut microenvironment and muscle-derived factors (myokines) (Gui et al., 2025). This multi-level regulatory model provides novel insights into brain aging and a foundation for designing targeted interventions.

As a non-pharmacological intervention, exercise modulates gut microbiota diversity and the production of metabolites such as SCFAs and neurotransmitter precursors. These metabolites influence intestinal barrier function and, via the circulation, affect central nervous system inflammation and neuronal function (Pinho et al., 2024). Concurrently, exercise promotes the secretion of myokines, some of which may support neuronal function and plasticity. Muscle-derived BDNF, for example, is thought to contribute to these processes, although its precise role is an area of active investigation. These interconnected effects form the biological basis of the brain-gut-muscle axis in regulating brain health. It is important to note that most mechanistic insights into the brain-gut-muscle axis are derived from preclinical models. Species-specific differences in gut microbiota composition (e.g., higher Firmicutes/Bacteroidetes ratio in mice vs. humans) and brain metabolism (e.g., rodent vs. human hippocampal neurogenesis rates) limit direct extrapolation to aging humans (Liu et al., 2026). This approach offers new perspectives for healthy aging and encourages a shift from single-target therapies toward systemic, biological regulation. Future research should prioritize interdisciplinary collaboration, integrating neuroscience, microbiology, exercise medicine, and nutrition to advance the translational application of interventions targeting this axis.

### 8.1 Critical appraisal of conflicting evidence and methodological limitations

Despite the growing body of evidence supporting the role of the brain-gut-muscle axis in exercise-mediated brain aging protection, several conflicting findings and methodological limitations in the literature warrant critical discussion.

#### 8.1.1 Conflicting findings

First, inconsistencies exist regarding the impact of resistance training on gut microbiota. While some studies report no significant changes in gut microbial diversity or composition following resistance training (Gao and Zhang, 2024), others have observed increased abundance of SCFA-producing bacteria (e.g., Bacteroides) in older adults after 12 weeks of resistance exercise (Wang Y. et al., 2025). This discrepancy may be attributed to differences in exercise intensity (e.g., moderate vs. high intensity), duration (short-term vs. long-term), and participant characteristics (e.g., baseline fitness, age, comorbidities). Second, conflicting results have been reported on the neuroprotective effects of specific myokines. For instance, while most studies demonstrate that irisin reduces cerebral β-amyloid accumulation in Alzheimer’s disease models (Lourenco, 2024), a recent preclinical study failed to observe this effect, possibly due to differences in animal strains, exercise protocols, or irisin detection methods (Kim et al., 2025). Third, the relationship between exercise-induced SCFA production and cognitive function remains inconsistent in human studies: some report a positive correlation between butyrate levels and memory improvement (Erlandson et al., 2021), while others show no significant association (Zhu et al., 2020), likely reflecting variations in dietary fiber intake (a key substrate for SCFA synthesis) and baseline gut microbiota composition across cohorts. While preclinical studies demonstrate that SCFAs cross the blood-brain barrier to inhibit neuroinflammation (Xiao et al., 2025), human data on SCFA concentrations in the cerebrospinal fluid (CSF) are scarce. Future clinical studies should measure CSF SCFA levels to validate this mechanistic pathway in aging populations.

#### 8.1.2 Methodological limitations

Existing studies also suffer from several methodological constraints. First, preclinical studies often use young, healthy animal models, which may not fully recapitulate the physiological changes associated with aging (e.g., immunosenescence, gut dysbiosis), limiting the translational relevance of their findings (Caruso et al., 2024). Second, clinical studies frequently have small sample sizes (< 50 participants) and lack long-term follow-up, making it difficult to draw definitive conclusions about the sustained effects of exercise on the brain-gut-muscle axis (Wang et al., 2021; Zhu et al., 2020). Third, exercise protocols (e.g., type, intensity, duration) are highly variable across studies, with no standardized approach, leading to inconsistent results (Gao and Zhang, 2024; Wang Y. et al., 2025). Fourth, gut microbiota analysis methods vary (e.g., 16S rRNA sequencing vs. metagenomics), and myokine detection techniques (e.g., ELISA vs. mass spectrometry) differ in sensitivity and specificity, further contributing to literature inconsistencies (Gao et al., 2024; Tabone et al., 2021). Finally, most studies fail to control for confounding factors such as diet, sleep quality, and medication use, which can independently modulate gut microbiota and brain function (Nandwana et al., 2022).

#### 8.1.3 Future directions to resolve inconsistencies

To address these limitations, future research should prioritize: (1) multicenter, large-sample clinical trials with standardized exercise protocols (e.g., duration, intensity, frequency) to ensure reproducibility; (2) long-term follow-up studies to evaluate the sustained effects of exercise on the brain-gut-muscle axis in aging populations; (3) integration of multi-omics technologies (e.g., metagenomics, transcriptomics, metabolomics) to comprehensively characterize molecular mechanisms; (4) strict control of confounding factors (e.g., dietary fiber intake, sleep) to isolate the independent effects of exercise; and (5) comparative studies across different age groups, sexes, and disease states to account for individual variability.

### 8.2 Individual variability and personalized interventions

#### 8.2.1 Age-related variability

Aging significantly modulates the brain-gut-muscle axis response to exercise. In young adults (18–30 years), short-term moderate-intensity exercise (8 weeks) increases SCFA production by 25–30% and irisin secretion by 30–35% (Cataldi et al., 2022; Gao et al., 2024) with rapid restoration of gut microbial diversity post-exercise. In middle-aged adults (40–60 years), longer exercise duration (≥ 12 weeks) is required to achieve similar effects (SCFA increase: 20–25%), likely due to age-related declines in gut microbial stability and muscle mass (Erlandson et al., 2021; Zhu et al., 2020). In older adults (≥ 65 years), exercise-induced benefits are further attenuated: moderate-intensity aerobic exercise for 24 weeks increases BDNF by only 15–20% (vs. 23–28% in middle-aged adults) (Peng et al., 2024), and gut microbiota shifts (e.g., Akkermansia enrichment) are observed only in adults with preserved muscle mass (sarcopenia-free) (Yu et al., 2025; Zhu et al., 2020). Frail older adults may require combined exercise (aerobic+resistance) plus probiotic supplementation to achieve meaningful improvements in cognitive function (Sánchez et al., 2022).

#### 8.2.2 Sex differences

Sex-specific hormonal and physiological differences influence axis responses. In males, high-intensity exercise increases serum IL-6 by 40–50% (Bettariga et al., 2024), which synergizes with testosterone to promote muscle protein synthesis and SCFA production. In females, estrogen enhances gut barrier integrity, leading to 12–18% higher butyrate levels post-exercise compared to males (Imdad et al., 2024; Wang Y. et al., 2025). However, post-menopausal females (low estrogen) exhibit reduced gut microbial diversity and blunted irisin secretion (15–20% lower than pre-menopausal females) (Bettariga et al., 2024; Ha et al., 2025) requiring higher exercise frequency (5 vs. 3 days/week) to mitigate neuroinflammation (Peng et al., 2024). Additionally, female endurance athletes are more susceptible to gut dysbiosis from excessive high-intensity exercise (Morishima et al., 2021), emphasizing the need for sex-tailored intensity regulation.

#### 8.2.3 Baseline fitness

Baseline fitness strongly predicts exercise responsiveness. Sedentary individuals show greater gut microbiota shifts (beneficial bacteria increase by 30–35%) and myokine secretion (irisin: 35–40% increase) after 12 weeks of exercise compared to physically active individuals (10–15% increase) (Gao et al., 2024; Zhu et al., 2020). Physically fit older adults (≥ 150 min/week of moderate exercise) maintain higher BDNF levels and gut microbial diversity, requiring only maintenance exercise (2–3 days/week) to preserve cognitive function (Bilski et al., 2020). In contrast, sedentary individuals with baseline gut dysbiosis may need prebiotic supplementation (dietary fiber: 25–30 g/day) alongside exercise to enhance SCFA production (Nandwana et al., 2022).

#### 8.2.4 Comorbidities

Chronic comorbidities attenuate exercise-induced benefits. Older adults with type 2 diabetes show 50–60% lower exercise-induced SCFA production due to gut barrier dysfunction (Imdad et al., 2024; Zhang L. et al., 2022) and require combined resistance training+aerobic exercise to improve insulin sensitivity and neuronal function (Shen et al., 2024). Individuals with mild cognitive impairment (MCI) exhibit blunted BDNF responses to resistance training alone (Ruiz-González et al., 2021), but combined exercise+Bifidobacterium supplementation increases BDNF by 28% and improves memory scores by 20% (Peng et al., 2024; Wang Y. et al., 2025). Patients with neurodegenerative diseases (e.g., Alzheimer’ s disease) may benefit from low-intensity aerobic exercise (50–60% MHR) to avoid gut barrier disruption and LPS-induced neuroinflammation (Burtscher et al., 2021; Cantón-Suárez et al., 2024).

#### 8.2.5 Genetic background

Genetic polymorphisms influence axis responsiveness. The FNDC5 rs16835198 polymorphism (associated with reduced irisin secretion) is present in 20–25% of older adults, who show 30–40% lower exercise-induced BDNF expression (Ha et al., 2025; Lourenco, 2024). These individuals may require higher exercise intensity (70–75%MHR)+polyphenol supplementation to enhance neuroplasticity (Stull et al., 2024). Polymorphisms in GPR43 (SCFA receptor) reduce SCFA-mediated anti-inflammatory effects in 15–20% of populations (Sun et al., 2025), necessitating increased dietary fiber intake (30–35 g/day) to compensate. Additionally, APOE ε4 carriers (at higher risk for Alzheimer’ s disease) exhibit reduced gut microbial diversity and require longer exercise duration (≥ 24 weeks) to delay cognitive decline (Kim et al., 2025; Liu et al., 2026).

#### 8.2.6 Personalized intervention recommendations

Based on individual variability, we propose tailored strategies:Healthy older adults (65–75 years, sarcopenia-free): Moderate-intensity aerobic exercise (30 minutes/day, 5 days/week, 60–70% MHR) + dietary fiber (25 g/day) →targets SCFA production and BDNF enhancement.Post-menopausal females: Combined aerobic + resistance training (3+2 days/week) + probiotics (Bifidobacterium, 10t^0^ CFU/day) s (Bifidobacterium, 10ts SCFA production and BDNF enhancement.Post-menopausal females: Combined aerobic g ulate th days/week) +low-intensity aerobic exercise (2 days/week) + prebioticsty aerobic exercise (2s SCFA production and BDNF enhanc MCI patients (APOE ε4 non-carriers):Combined exercise (4 days/week) + anthocyanin-rich nutritionxercise (4CFA production and BDNF enhancementε4 carriers): Long-term moderate exercise (≥ 24 weeks) + BDNF-promoting probioticsd delays cognitive decline.
